# Supplementary material for: Enhancing haptic continuity in virtual reality using a continuity reinforcement skeleton
Source: Nat Commun. 2025 Mar 27;16:2995. doi: 10.1038/s41467-025-58318-z (PMC11950344; doi:10.1038/s41467-025-58318-z)
Supplement: Supplementary file 1 — Supplementary Information [file 41467_2025_58318_MOESM1_ESM.pdf]

Supplementary Information

Enhancing Haptic Continuity in Virtual Reality using a Continuity Reinforcement Skeleton

Xinyuan Wang [1], Zhiqiang Meng [1], and Chang Qing Chen [1, 2, *]

[1] Department of Engineering Mechanics, CNMM and AML, Tsinghua University

Beijing 100084, P.R. China

[2] Mechano-X Institute, Tsinghua University, Beijing 100084, PR China

* E-mail: chencq@tsinghua.edu.cn

Supplementary Note 1 Estimation of elongation of 2D haptic device

As mentioned in the main text, there is a trade-off between actuation travel and pixel size in 2D wearable haptic devices. To validate this theory, Supplementary Fig. 1a shows a simplified model to estimate the elongation in haptic devices. The size of the deformable part is d, and the off-plane displacement of the pixel is h. Then, the tensile strain can be estimated by the following equation

$$\varepsilon_{Esti} = \left[\frac{\sqrt{h^2 + \left(d/2\right)^2}}{d/2} - 1 \right] \times 100\% \quad (1)$$

The relationship between ε_{Esti} and d/h is shown in Supplementary Fig. 1. Once d/h is reduced below 2, the elongation required to produce the same off-surface displacement rises sharply with the decrease of the cell size. The material can withstand a finite amount of tensile strain, constraining the ratio between cell size d and travel distance h.

Supplementary Note 2 Definition and empirical formula for continuity

According to the definition of D_S and D_P for 1D cases in the main text, the expectation can be estimated by calculating the averaged distortion during the movement. Supplementary Fig. 2a shows the surface shape of a general 1D haptic device. Let the green dashed line on the left side be the reference position and coincide with a pixel point. When the ideal wave peak and the reference position coincide, the ideal peak position is $x_i = 0$. The distance between the green dashed lines is the pixel pitch d. The following formulae can calculate the instantaneous distortion

$$d_P = \frac{\left| x_r - x_i \right|}{l} \quad (2)$$

$$d_S = \sqrt{\frac{\int_{x_i - l/2}^{x_i + l/2} \left(\varphi(x, x_i) - \psi(x, x_i) \right)^2 \mathrm{d}x}{\int_{x_i - l/2}^{x_i + l/2} \left(\varphi(x, x_i) \right)^2 \mathrm{d}x}} \quad (3)$$

in which l is the wavelength, x_r is the actual peak position, and $\varphi(x, x_i)$ is the ideal waveform defined by

$$\varphi(x, x_i) = \frac{h}{2} + \frac{h}{2}\cos\frac{2\pi(x - x_i)}{l} \tag{4}$$

where $x_i - l/2 \le x \le x_i + l/2$. $\psi(x, x_i)$ is the actual waveform.

Supplementary Fig. 2b illustrates the distortion of square pixels. The green dashed line on the left side represents the reference location. At this point, x_r coincides with the reference pixel point, i.e. $x_r = 0$, and d_p is equal to x_i/l. The blue part represents the residual between the ideal and actual waveforms and d_S can be calculated from Supplementary Equation (3). Set $x_i = 0$ when x_i coincides with x_r, $l = 1$. The relation of d_S and d_P with x_i/d is shown in Supplementary Fig. 2c and d when $d/l = 1/3$. $\overline{d_S}$ and $\overline{d_P}$ for a given value of d/l is obtained by taking the mean of the distortions in one period. The curve of mean distortion with d/l can be obtained by varying the wavelength l, as shown in Supplementary Fig. 3. By fitting the datasets, the empirical formulas of the shape distortion of pixel and linear connection is $D_S = 0.994\,d/l$ and $D_S = 1.545(d/l)^2$, respectively, and the corresponding R^2 is marked under the graph. On the other hand, the D_P can be derived from definitions theoretically by integrating d_P in the part between pixels and then dividing by the length of this part. For 1D pixel and linear connection, it is

$$D_P = \int_{-d/2}^{d/2} \frac{|x|}{dl}\,\mathrm{d}x = 0.25\,d/l \tag{5}$$

For the 2D case, the average distortion can be similarly obtained and fitted. The equation for the instantaneous distortion in 2D is given by

$$d_P = \frac{\sqrt{(x_r - x_i)^2 + (y_r - y_i)^2}}{l} \tag{6}$$

$$d_S = \sqrt{\frac{\iint\limits_S \left(\varphi(x, y, x_i, y_i) - \psi(x, y, x_i, y_i)\right)^2 \mathrm{d}x\mathrm{d}y}{\iint\limits_S \left(\varphi(x, y, x_i, y_i)\right)^2 \mathrm{d}x\mathrm{d}y}} \tag{7}$$

The ideal waveform is

$$\varphi(x,y,x_i,y_i)=\frac{h}{2}+\frac{h}{2}\cos\frac{2\pi\sqrt{\left(x-x_i\right)^2+\left(y-y_i\right)^2}}{l} \tag{8}$$

where $\left(x,y\right)\in S$, $S=\left\{\left(x,y\right)\mid\left(x-x_i\right)^2+\left(y-y_i\right)^2\le l^2/4\right\}$, and $\left(x_i,y_i\right)$ is the ideal peak position. The distortions of the square and hexagonal pixels are analyzed in Supplementary Fig. 4. For square pixel, $D_S=1.412\,d/l$; for hexagon pixel, $D_S=1.318\,d/l$. Here, D_P can also be derived theoretically by double integrating d_P in the region between pixels and dividing by the region's area because of the uniform distribution assumption. For square pixels, it is

$$D_P=\int_{-d/2}^{d/2}\int_{-d/2}^{d/2}\frac{\sqrt{x^2+y^2}}{d^2 l}\mathrm{d}x\mathrm{d}y=0.3825\,d/l \tag{9}$$

For hexagonal pixels, it is

$$D_P=\int_0^{\sqrt{3}d/2}\int_0^{\sqrt{3}x/3}\frac{\sqrt{x^2+y^2}}{\frac{\sqrt{3}}{24}d^2 l}\mathrm{d}y\mathrm{d}x=0.3510\,d/l \tag{10}$$

Furthermore, the formula of D_P for 2D Continuity Reinforcement Skeleton (CRS) can also be obtained. Since the 2D CRS can display the real peak along the nearest interpixel line to the ideal peak, for square pixel, it can be obtained that

$$D_P=\int_0^{d/2}\int_0^{x}\frac{y}{\frac{1}{8}d^2 l}\mathrm{d}y\mathrm{d}x=\frac{1}{6}d/l \tag{11}$$

For hexagonal pixels, it is

$$D_P=\int_0^{d/2}\int_0^{\sqrt{3}x/3}\frac{y}{\frac{\sqrt{3}}{24}d^2 l}\mathrm{d}y\mathrm{d}x=\frac{\sqrt{3}}{18}d/l \tag{12}$$

In summary, the empirical formulas of D_S for different types of haptic devices can be obtained by linear fitting, and D_P is obtained from theoretical derivation.

Supplementary Note 3 Experimental measurement of distortion

To verify the effectiveness of CRS for continuity reinforcement, the surface displacement field of the haptic display device needs to be captured and analyzed. Here, we obtain the displacement field of the target surface using the DIC (Digital Image Correlation) method. For 1D CRS devices, the displacement field is obtained by spraying the skeleton with speckles and photographing it with a binocular DIC camera, shown in Supplementary Fig. 5a. However, many haptic devices have pixels that cannot fill the entire plane, making it challenging to derive their surface displacement. To address this, we use spandex textile with speckles to cover the haptic device, representing the surface displacement, as demonstrated in Supplementary Fig. 5. The displacement measurement method introduced here is experimentally based and applicable to most haptic devices.

The distortion can be obtained by comparing the target waveform with the surface displacement field information. Decoupling position distortion and shape distortion can bring more convenience to the measurement. The process of experimentally measuring the two types of distortion of a 1D haptic device is shown in Supplementary Fig. 6. The haptic device to be tested is first made to display a sinusoidal wave of a given wavelength and amplitude moving at a uniform speed. The surface deformation is obtained by the DIC method. Then the peak position can be found. By comparing the variation of the peak position with its target position, $\overline{d_P}$, or experimentally measured D_P, can be obtained by Supplementary Equation (2) and (6). When calculating shape distortion, to decouple the effect of peak position distortion, the experimentally measured instantaneous distortion d_S can be defined as

$$d_S = \min_{x_i} \sqrt{\frac{\int_{x_i - l/2}^{x_i + l/2} \left(\varphi(x, x_i) - \psi_E(x)\right)^2 \mathrm{d}x}{\int_{x_i - l/2}^{x_i + l/2} \left(\varphi(x, x_i)\right)^2 \mathrm{d}x}} \tag{13}$$

where ψ_E is the displacement field obtained from the DIC method and is independent of x_i. The d_S obtained in each frame is calculated and plotted as a function image at the bottom figure and averaged to obtain $\overline{d_S}$, the experimentally measured D_S. 2D haptic devices can be measured in the same way as the 1D devices, except that the formula is changed

$$d_S = \min_{(x_i, y_i)} \sqrt{\frac{\iint_S \left(\varphi(x, y, x_i, y_i) - \psi_E(x, y)\right)^2 \mathrm{d}x\mathrm{d}y}{\iint_S \left(\varphi(x, y, x_i, y_i)\right)^2 \mathrm{d}x\mathrm{d}y}} \tag{14}$$

According to the above equation, the distortion curves of 1D CRS for different d/l cases shown in Supplementary Fig. 7 can be obtained. The average distortion D_P and D_S are labeled at the bottom of each figure. Supplementary Fig. 8 illustrates the distribution of peaks of each frame for 2D haptic devices with and without CRS. The crosses in the figure represent the experimentally measured peaks, and the yellow arcs are the ideal wave peaks, which move with a radius of 30 mm. It can be seen that the peaks concentrate at a few pixel points without CRS. On the contrary, the wave peaks can move along the skeleton when equipped with CRS, improving continuity. Supplementary Fig. 9 shows the experimentally measured shape distortion of the 2D haptic device. At the same pixel density, CRS reduces the shape distortion by half.

Supplementary Note 4 Display resolution limits for CRS

When examining the resolution of the haptic device, shorter the wavelength allowed for displaying more haptic details can be displayed. However, as mentioned in Supplementary Note 2, under fixed pixel pitch d, shorter the wavelength lead to higher the distortion. Consequently, sharper edges are the more challenging to display. Although CRS can reduce distortion and enhance continuity without increasing pixel density, it is still necessary to discuss its minimal display resolution. To explore the minimal display wavelength of CRS, the 1D CRS distortions at higher d/l were

tested experimentally, as depicted in Supplementary Fig. 10. Notably, shape distortion rises significantly due to the insufficient constraints. In 1D, if the shape distortion does not exceeding 0.1, the resolution of the CRS is about $d/l = 0.3$, according to Supplementary Fig. 11.

To explore the limits resolution of the 2D CRS, the test in Supplementary Fig. 12a and b was designed. Noting that the distortion is related to the motion's path in 2D. To test the extreme situation, the radius of motion is 15mm, half of the pixel pitch, because this peak moving track is furthest from the pixel point. Supplementary Fig. 12c shows the distortion curve for this case. The CRS can still reduce the shape distortion by half. In 2D, if the shape distortion does not exceeding 0.2, the resolution of the CRS is about $d/l = 0.3$.

Supplementary Note 5 Mechanical Model for Continuity Reinforcement Skeleton

According to the previous section, the smallest wavelength that a CRS can display is approximately three times the pixel pitch. In this section, the mechanics of CRS are analyzed only for the case of $d/l = 1/3$. The first reason for analyzing this situation is that CRS is often required to display features with as small wavelengths as possible. The second reason is that the effect of collapse on haptics is more significant at small d/l.

When the wave peak moves to the pixel point, it does not collapse easily due to the support of the pixel point. Therefore, for the subsequent analysis of the no-collapse condition, the situation when the wave peak moves to the midpoint of the two pixels was selected for analysis.

Supplementary Fig. 13a illustrates the CRS ($d/l = 1/3$) in contact with silicone. For ease of analysis, the silicone is simplified to a Winkler foundation, in which the linearly distributed force q of the foundation on the CRS is proportional to the

deflection of the beam. The following relation is satisfied

$$q = \beta y(x) \tag{15}$$

in which β is the 1D coefficient of the subgrade reaction of the Winkler model. Here, β is determined by the stiffness of silicones and also affected by the skeleton's width. According to M. Hetenyi's theory of the deformation of beams on elastic foundation, the deflection equation of the beam $y(x)$ in Supplementary Fig. 13b has the following series form

$$y(x) = 4Pl^3 \sum_{n=1,2,3...}^{\infty} \frac{\left[1-\cos\frac{n\pi(l-d)}{l}\right]\left(1-\cos\frac{2n\pi x}{l}\right)}{n^4 16\pi^4 EI - 4n^2\pi^2 l^2 N + 3\beta l^4} \tag{16}$$

Here, l is the wavelength and $l = 3d$, n is the wave number of the deformation, N is the axial force, E is the Young's modulus of the beam, I is the moment of inertia of the beam's cross-section, and P is the support force from pixels. Let $n^4 16\pi^4 EI - 4n^2\pi^2 l^2 N + 3\beta l^4 = 0$, the critical axial force $N_{cr}^{(n)}$ for mode buckling at a wavelength of l/n can be obtained

$$N_{cr}^{(n)} = \frac{n^4 16\pi^4 EI + 3\beta l^4}{4n^2\pi^2 l^2} \tag{17}$$

It can be seen from Supplementary Equation (17) that $N_{cr}^{(n)}$ does not vary monotonically with n. When $N_{cr}^{(1)} < N_{cr}^{(3)}$, the axial force will induce a deflection mode with $n=1$, presenting a complete sinusoid so that it will not collapse. Otherwise, if the deflection mode has a wavelength equal to the pixel pitch, i.e. $n=3$, the beam is no longer constrained by pixels anymore, resulting in collapse. Based on this analysis, we can obtain the no-collapse condition

$$\Delta = \frac{16\pi^4 EI}{27\beta d^4} > 1 \tag{18}$$

The experiment shown in Supplementary Fig. 13a was conducted to validate this condition, utilizing steel beams of equal width but different thicknesses and lengths

pressed into silicone of varying hardness. There are three kinds of beams made of different material: 301 stainless steel ($E = 193\text{GPa}$), Al 1060 ($E = 69.5\text{GPa}$), and Copper H62 ($E = 105\text{GPa}$). The above metal labels are in accordance with GB standards. Three thicknesses (0.1mm, 0.15mm, and 0.2mm) and three pixel pitches (10mm, 17mm, and 20mm) were also tested. The silicones and beams used here is demonstrated in Supplementary Fig. 14. I can be obtained from the formula, $I = ba^3/12$, where b is the beam width and a is the thickness. We also modulated three different hardnesses of silicone. Silicone 1: Eco-flex 00-30; Silicone 2: Eco-flex 00-30, which contains 33.3% silicone thinner by mass; Silicone 3: Eco-flex 00-10. Their mechanical properties were measured by the tests shown in Supplementary Fig. 13c and d. The indenter in the figure uses the same width as the steel beam (4 mm) and is flanked by sinusoidal waves with wavelengths of 30 mm, 51 mm and 60 mm, and an amplitude of 8 mm. The force-displacement curves resulting from the compression of the three silicones with three different sinusoidal-wavelength-shaped indenters are shown in Supplementary Fig. 15. The corresponding silicone numbers and wavelengths of the indenter shapes are labeled below the image.

According to Winkler foundation assumption, the generated force $F_{\max}$ when the indenter is fully pressed into the silicone has the following relationship with β

$$\beta = \frac{F_{\max}}{S_{\text{sine}}} \tag{19}$$

This equation can be obtained by integrating the Winkler Foundation's definition Supplementary Equation (15). The corresponding β of Supplementary Fig. 15 is shown in Supplementary Table 1. Due to the simplified treatment of the mechanical properties of elastomers in the Winkler foundation, the β is calculated separately for different wavelengths of the indenter. The phase diagram in main text Fig. 3d was then plotted based on the experimental results.

Based on above analysis, a natural question is what will happen if $\Delta \approx 1$. The experiment in Supplementary Fig. 16 was conducted. We used a steel beam with a thickness of 0.2 mm and a total wavelength of 60 mm to press into silicone 1, where $\Delta = 1.0243$. Whether the beam will collapse depends on its status when it is pressed in. If it is initially pressed in as a no-collapsed shape, it will not collapse, as shown in Supplementary Fig. 16a; conversely, if the beam is pre-collapsed and then pressed in, it will remain collapsed, as shown in Supplementary Fig. 16b. This phenomenon may come mainly from the friction on the interface, which hinders the transformation between collapse and no-collapse.

It is also desirable to solve the support reaction of the boundary servos and pixel servos. Taking the first two terms of the serial solution in Supplementary Equation (16), we get

$$y = 4Pl^3 \left[k_1 \left(1 - \cos\frac{2\pi x}{l} \right) + k_2 \left(1 - \cos\frac{4\pi x}{l} \right) \right] \tag{20}$$

in which

$$k_2 = \frac{3}{512\pi^4 EI - 288\pi^2 d^2 N + 486\beta d^4} \tag{21}$$

However, because $N = N_{cr}^{(1)} = \left(16\pi^4 EI + 3\beta l^4\right) / 4\pi^2 l^2$ is obtained by letting $n^4 16\pi^4 EI - 4n^2\pi^2 l^2 N + 3\beta l^4 = 0$, the value of k_1 should be determined by the geometric constraints. The compression of the boundary servos should be $\Delta L = \Delta l + \Delta L_0$, in which Δl is the reduction in the total length of the beam due to the axial force, and ΔL_0 is the length increment due to the off-plane deformation. Note that when $\Delta \gg 1$, $\Delta l = N_{cr}^{(1)} L / EA \approx \pi^2 b^2 L / 3l^2$ is material-independent, with L and b being the length and thickness of the beam, respectively. The change in beam length Δl is negligible for CRS with high Young's modulus and small thickness. Considering the constraints of the support points and servos compression on both sides, we get

$$\begin{cases} \Delta L \approx \frac{1}{2}\int (y')^2 dx = 16\pi^2 P^2 l^5 \left(k_1^2 + 4k_2^2\right) \\ \frac{3}{4}h = 4Pl^3\left[k_1\left(1-\cos\frac{2\pi}{3}\right) + k_2\left(1-\cos\frac{4\pi}{3}\right)\right] \end{cases} \quad (22)$$

The equation is simplified to obtain

$$\begin{cases} \Delta L = 16\pi^2 P^2 l^5 \left(k_1^2 + 4k_2^2\right) \\ h = 8Pl^3\left(k_1 + k_2\right) \end{cases} \quad (23)$$

The following results on the pressure on the pixel point and the shape of the deformed beam can be obtained by combining Supplementary Equations (21), (22), and (23).

$$\begin{cases} N = N_{cr}^{(1)} = \dfrac{16\pi^4 EI + 243\beta d^4}{36\pi^2 d^2} \\ k_2 = \dfrac{3}{512\pi^4 EI - 288\pi^2 d^2 N + 486\beta d^4} \\ k_1 = \dfrac{12d\Delta L + 2\pi h\sqrt{15d\Delta L - \pi^2 h^2}}{\pi^2 h^2 - 12d\Delta L} k_2 \\ P = \dfrac{h}{216d^3\left(k_1 + k_2\right)} \end{cases} \quad (24)$$

Supplementary Fig. 17 shows the relationship between the reaction force and the compression of boundary servos, with $E = 210\text{GPa}$, $\beta = 210\text{kPa}$, $d = 10\text{mm}$, and $h = 10\text{mm}$. When using the direct control method to determine ΔL (see Supplementary Note 7), the reaction force on the pixels is shown as the circle in orange. If the actuator of the haptic device is force-controlled, we can use Supplementary Equation (24) to calculate the amount of required force to achieve a specific target shape.

Supplementary Note 6 Assembling of Continuity Reinforcement Skeleton

The several types of CRS depicted in the article are all assembled from basic units. The units can be mainly classified into internal units and boundary units. The internal units regulate the height of the pixels, while the boundary units control the amount of compression of the skeleton.

Supplementary Fig. 18 illustrates the assembly process of the internal unit. First,

the support is fixed on the servo, and then the servo is fixed to the base. Supplementary Fig. 19 illustrates the composition of the boundary cells. The boundary servos move horizontally. The support is connected to the servo and is constrained in the track. A detail of the connection between the boundary unit and the skeleton is shown in Supplementary Fig. 20a. The forked design of the end of the skeleton is shown in Supplementary Fig. 20b. Supplementary Fig. 20c shows the support of the internal unit, with the fishing line protruding through the hole at the top to constrain the skeleton. A photo of the linear servo used in this study is shown in Supplementary Fig. 20d.

Supplementary Note 7. Control Algorithms of Continuity Reinforcement Skeleton

To control the CRS display surface, it is necessary to calculate the travel distance of the boundary servos. The key problem is to calculate the compression of each CRS for a given specific pattern. Two methods have been proposed to perform the calculation: the direct control method and the interpolation control method.

Supplementary Note 7.1 Direct Control Method

The direct control method involves calculating the travel distance of the boundary servos, based on the surface equations. This method is suitable for surfaces that can be described by a simple analytical equation. In this paper, all the CRS experiments are controlled using this method.

Consider a surface $z=\varphi(x,y)$ with a CRS beam placed on the xOy plane. The projection of the CRS onto the plane is given by $y=y_l(x)$. The start and end points of the display area of the CRS beam are x_s and x_e, respectively. Denoting the respective original and deformed lengths of the CRS beam within the display area by L_0 and L_t, the total travel distance ΔL for the servo motors on this CRS beam is given by

$$\Delta L = L_t - L_0 = \int_{x_s}^{x_e} \sqrt{1 + \left(\frac{dy_l}{dx}\right)^2 + \left(\frac{\partial z}{\partial y_l}\frac{dy_l}{dx} + \frac{\partial z}{\partial x}\right)^2}\, dx - L_0 \tag{25}$$

It should be noted that the above integral expression does not always have an analytical solution. A more effective approach to calculating the length of a curve is to discretize it as a polyline, as a first-order approximation. Let there be a list of uniformly distributed points on the curve $\left(x_j, y_j, z_j\right)$, in which $j = 1, 2, ..., n$. For planar CRS problems, the above list of points can simply be generated at equal intervals in the x-direction. The distance ΔL can then be estimated by

$$\Delta L = L_t - L_0 = \sum_{j=1}^{n-1} \sqrt{\left(x_{j+1} - x_j\right)^2 + \left(y_{j+1} - y_j\right)^2 + \left(z_{j+1} - z_j\right)^2} - L_0 \tag{26}$$

Note that unlike the previous formulas in integral form, the above calculation of lengths does not require derivation. This formula is also applicable to CRS on curved surfaces if the discretization is done in a reasonable way. It should be noted that the direct control method is only suitable if the equation of the CRS is known, which is often not available in practical VR environments.

Supplementary Note 7.2 Interpolation Control Method

If the equations of the surface are unknown and only the heights at the pixel points are available, the travel distance of the boundary servos can be approximated using the third-order Hermite interpolation, a method for segmental interpolation using cubic functions while ensuring second order continuity at the nodes. Its interpolation function takes the form

$$y_i\left(x\right) = a_i x^3 + b_i x^2 + c_i x + d_i \tag{27}$$

where $y_i\left(x\right)$ is the interpolation function for the segment from $\left(x_i, y_i\right)$ to $\left(x_{i+1}, y_{i+1}\right)$. For data with n points, the coordinates of data points provide $2n$ equations, the first and second order continuity conditions provide $2n-2$ equations, and the remaining two equations are given by the boundary slopes.

The curvature of a beam under small deformations can be approximated by the second order derivative of its displacement $z''(x)$ and is proportional to the bending moment $M(x)$, i.e. $z''(x) = M(x)/EI$ where EI is the bending stiffness. In the CRS, $M(x)$ is continuous when there is no concentrated bending moment. Based on the above analysis, the use of third-order Hermite interpolation to calculate the compression of the boundary servos is a reasonable approximation, because this method guarantees $z \in C^2[x_s, x_e]$.

Next, we discuss the problems faced when using the interpolation control method and their solutions. Starting with a 1D case, let the deformation equation of the CRS be Supplementary Equation (4), i.e., $z(x) = \varphi(x, x_i) = h\left[1 + \cos 2\pi (x - x_i)/l\right]/2$, in which $x_i - l/2 \le x \le x_i + l/2$, h is the wave amplitude, l is the wave length, and x_i is the peak position. Let the pixel spacing be d. When the displayed wavelength is close to the pixel point spacing, the interpolation function does not fit the target waveform well due to the low sampling rate, shown in the upper part of Supplementary Fig. 21a. As a result, a biased estimate of ΔL is obtained, as shown in Supplementary Fig. 21b when the wave peak is moving between pixels. The effect of the normalized display wavelength l/d and normalized amplitude h/d on the prediction error of ΔL is analyzed. Define the estimation error rate of ΔL as

$$e_{\Delta L} = \frac{\int_0^d \left|\Delta L_t - \Delta L_e\right| dx_i}{\int_0^d \Delta L_t dx_i} \times 100\% \tag{28}$$

where ΔL_t is the target compression and ΔL_e is the estimated compression calculated from the interpolation curve. This definition can be considered as the ratio of the area of the red portion to the portion below the blue straight line in Supplementary Fig. 21b. The error surface of the interpolation algorithm for different normalized wavelengths and amplitudes can be obtained by numerical calculations, as shown in Supplementary

Fig. 21c. It can be seen that the error becomes evident for low amplitudes and short wavelengths.

The minimum normalized wavelength that the CRS can display in this paper is $l/d = 3$. Accurate estimation of ΔL at this wavelength is important. According to the previous analysis, the interpolation algorithm produces an estimation error of about 10% when $h/d = 1$, as shown in the left panel of Supplementary Fig. 21d. When higher accuracy is needed, it is necessary to increase the rendering resolution in the controller memory. For instance, in the lower part of Supplementary Fig. 21a, the number of pixels in this array is doubled, and an auxiliary rendering node is added between each pair of pixel points to more accurately calculate the amount of compression. In the right panel of Supplementary Fig. 21d, the corresponding error of compression is shown to have reduced to one tenth. In two dimensions, this algorithm requires four times the amount of computation, which is often affordable considering the small array size of existing haptic displays compared to computer screens.

For wearable devices with the CRS, the total length of the beam on a spatial curve is often required. For instance, the blue spatial curve shown in Supplementary Fig. 22a, with its projection on xOy and yOz represented by black lines, consists of four independently controlled pixel points. The arc length s can be used as a parameter to represent an arbitrary spatial curve, with the arc length corresponding to the data points defined as s_i for each data point. Subsequently, $x_i \sim s_i$, $y_i \sim s_i$, $z_i \sim s_i$ are independently interpolated as shown in Supplementary Fig. 22b. With the arc length parameter s, the directional vector of the curve at each point is given by $(dx/ds, dy/ds, dz/ds)^T$. Interpolation curves can be uniquely determined by considering the tangent direction conditions at the start and end points as shown in Supplementary Fig. 22c by a red curve. To use this method, it is necessary to calculate

the arc length s_i of the pixel point on each skeleton when it is not deformed. s_i for each pixel do not change after deformation by noting that the length change of the skeleton is negligible compared to its whole length. If increased control accuracy of the boundary servos is desired, a double rendering resolution is recommended. To facilitate the understanding of the three compression calculation methods in this paper, we listed their advantages and disadvantages in Supplementary Table 2.

Supplementary Note 8 Mechanical limitation of Continuity Reinforcement Skeleton

Although CRS can enhance the display continuity of a haptic device, it also introduces additional motion constraints. These mechanical limitations arise mainly from the travel of the boundary servos and the limitations of the beam material, which are discussed separately below.

In the previous section, a method for controlling the compression of CRS boundary servos was provided. When both ends of the servos are set, the range of variation for the CRS length cannot be greater than twice the servo travel $u_{\max}$, that is

$$\max\left(\Delta L_e\right)-\min\left(\Delta L_e\right)\leq 2u_{\max} \tag{29}$$

When designing the CRS system, it is important to determine the maximum and minimum lengths of the CRS with a known pixel spacing d, maximum pixel travel h, and the shape of the base. Due to the complexity of interpolating or solving the real CRS motion model, it is difficult to determine the pattern corresponding to $\max\left(\Delta L_e\right)$ and $\min\left(\Delta L_e\right)$. In the following, we present an approximate estimate to facilitate the design.

Consider the extreme case of $h \gg d$. For planar bases, the shortest and longest situations are shown in Supplementary Fig. 23a and b, respectively. A CRS with $2n+1$ pixel points requires a boundary servo travel distance of about $2nh$. And for a circular

base with $2n$ pixels whose radius is r, the shortest and longest situations are shown in Supplementary Fig. 23c and d. The required travel of boundary servo here is about $2nh+2\pi h$.

Based on the above analysis, we can assess the feasibility of equipping CRS on the human body. The radius of the lower arm of an adult male is about 40mm. Around the arm, we set 39 pixels, with a pitch of about 6mm. Assuming a maximum height of 3 mm for each pixel, we can use the interpolation method to calculate the required action distance. Compared to the length of CRS with all pxiel heights at 0, the shortest CRS requires the boundary servo to stretch by 19 mm, while the longest CRS requires the boundary servo to compress by 35mm (Supplementary Fig. 23e and f). Considering that there are two servos for each skeleton, the total travel distance required for a single servo is 27mm, which is an acceptable value.

The yield strength of the beam material restricts the maximum bending curvature of the skeleton. According to the formula for the bending normal stress in the cross-section of a beam, when the yield stress of the material is σ_u, its curvature κ at any point cannot exceed

$$\kappa<\frac{\sigma_u}{Ea} \tag{30}$$

where E is the Young's modulus of CRS, and a is the thickness. Based on the interpolation of the pixel, κ at every point can be calculated by

$$\kappa(s)=\left|\frac{\vec{r_i}'(s)\times\vec{r_i}''(s)}{\vec{r_i}'(s)}\right| \tag{31}$$

where $\vec{r_i}(s)=(x_i,y_i,z_i)$ is the interpolation equation of the segment i, and $\vec{r_i}'(s)$ and $\vec{r_i}''(s)$ are the first and second order derivatives. With the above equation, the deformation condition of any CRS can be programmed to be monitored in real time in order to prevent plastic deformation of the CRS.

In summary, the mechanical limitations imposed by CRS can be estimated by Supplementary Equations (29), (30), and (31).

Supplementary Note 9 Haptic identification of handwritten digits

To investigate the impact of continuity on the haptic display, we measured and compared the accuracy of digit identification using palm haptic with and without CRS. In the experiment, volunteers were instructed to feel the movement of contact (similar to main text Fig. 5b) and identify the corresponding number of the trajectory, see Supplementary Fig. 24. The two haptic devices share the same shape and a pixel pitch of 30 mm. The shapes of the displayed digits from 0 to 9 are shown in Supplementary Fig. 25. For each number, we collected five different handwriting patterns. Here some of the numbers, such as 8 and 9, have different stroke orders, as shown in Supplementary Fig. 25. The numbers 4 and 5 will be written in two strokes, while all other numbers will be written in one stroke. These digits have an average width of 12.3 mm and an average height of 18.9 mm. Notice that the size of the numbers is much smaller than that in the VR demo. This design demonstrates that the CRS can accurately display the peak locations, even in the extreme case when the number size is smaller than that of the pixel pitch. The shape of the contact point is determined by Supplementary Equation (8), in which $h = 10\text{mm}$ and $l = 100\text{mm}$, while the peak position is determined by the digits' shape.

A total of eight volunteers (six males and two females) used their left hand for number identification. Each volunteer had 20 learning opportunities, i.e., the numbers were displayed according to the volunteers' request to facilitate their familiarization with the task. The volunteers then performed four sets of identification experiments, with each group recognizing ten random numbers (which could be repeated). Half of the volunteers tested the haptic device with CRS first, then measured pixel haptics,

while the other half did so in reverse order.

The confusion matrices shown in Supplementary Fig. 26 were plotted based on the volunteers' identification of numbers. The numbers in the blocks indicate the frequency of the identification results corresponding to specific ground truth. For instance, the "1" at the lower-left corner of the pixel confusion matrix means that the volunteers mistakenly answered "0" while displaying "9" one time. Blue blocks represent correct identification, while orange blocks represent incorrect ones. By improving the continuity of the haptic display, the accuracy in identifying numbers with similar strokes (such as "0" and "6", "7" and "9") increases. The average accuracy of the CRS device is 84.7%, while that of the pixel haptic is 69.9%. By enhancing motion continuity, the accuracy of user identification is improved by about 14.8%.

Supplementary Note 10 Pressure distribution on the skin

Note that measuring the pressure on the skin surface is often challenging, due to interference from accurate pressure sensors on the pressure field. However, the pressure distribution can be simulated using the FEM method. In order to calculate the pressure distribution, it is necessary to first measure the mechanical properties of the skin. As an example, we measured the uniaxial compressive stress-strain curve of silicone, as shown in Supplementary Fig. 27. Silicone was chosen to mimic the skin. The obtained Young's modulus was 63.9 kPa and the Poisson's ratio was about 0.25.

In the FEM simulations, the boundary conditions were set to match those in the CRS, with a maximum indentation depth of 3 mm into the skin. Other parameters include a beam width of 4 mm, thickness of 1 mm, and a sinusoidal shape with a wavelength of 51 mm. In order to validate the finite element model, the FEM predicted strain field is compared with the corresponding experimental measurement by the Digital Image Correlation (DIC) method. The strain fields presented in Supplementary

Fig. 28 show good agreement between simulations and experiments. The observed minor difference is attributed to the different locations of the pressure imposed by the two methods: the CRS is applied to the center of the skin in the FEM method, while the indenter is applied to the edges in the DIC method because the DIC method can only measure surface deformation.

The FEM predicted pressure fields on the skin surface, induced by the CRS with different Young's moduli, are shown in Supplementary Fig. 29. The pressure field remains unchanged when the modulus of the beams is increased to 10 and 100 times the original value, which indicates that the pressure field is independent of the material of the beams when the no-collapse condition is satisfied. Consequently, the CRS is displacement-controlled and insensitive to the choice of materials.

Supplementary Note 11 Latency measurement

Latency is essential for haptic devices. First, we assessed the latency of the CRS device. The MATLAB control program will display "start" on the screen after sending the control signal to the CRS device, allowing us to measure the latency by calculating the time difference between the appearance of "start" and the response of the CRS device, as shown in Supplementary Fig. 30a. The latency is 75ms. To test the latency of the VR-haptic system, we set the camera to high-speed mode and positioned the user and the haptic device within the same frame, as shown in Supplementary Fig. 30b. Initially, the user's hand rested at the center of the virtual character's hand, and then the user was instructed to start pressing. The delay time was measured by calculating the time difference between the start of the hand movement and the response of the haptic device. The latency in this case is 160ms.

List of figures

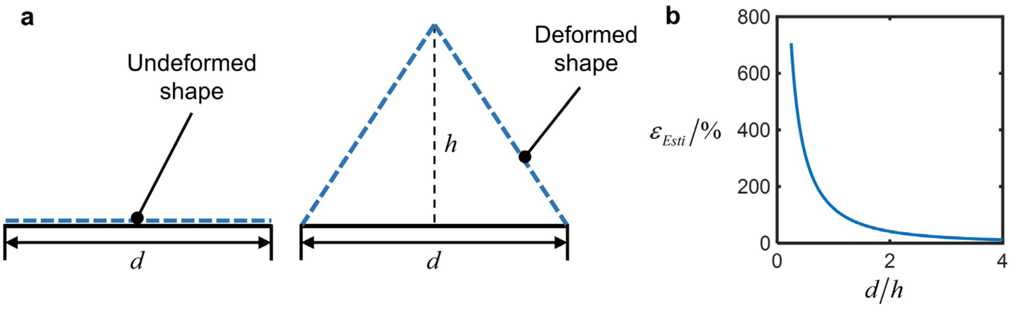

Supplementary Fig. 1 Trade-off of travel distance and pixel size in a 2D haptic device. a 2D haptic device model for elongation estimation. **b** Relationship between tensile strain and d/h.

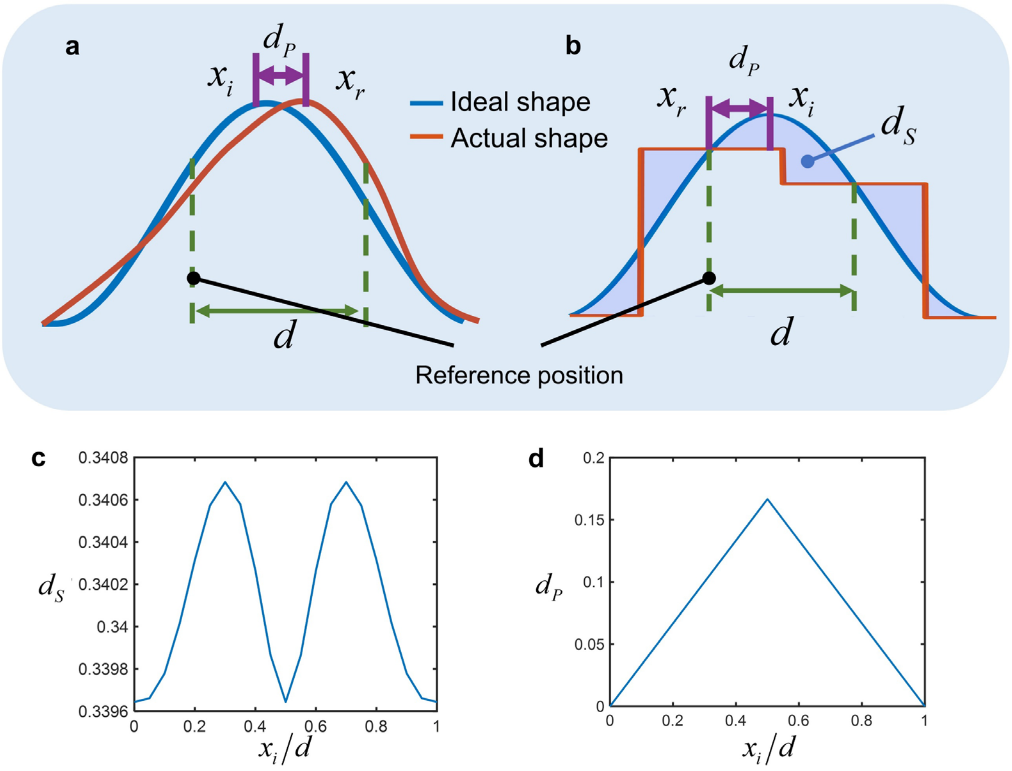

Supplementary Fig. 2 Instantaneous distortion of a haptic device. a Distortion definition of arbitrary haptic devices. **b** Distortion definition of a square pixel haptic device. **c** Instantaneous shape distortion curve of square pixel at $d/l = 1/3$. **d** Instantaneous position distortion curve of square pixel at $d/l = 1/3$.

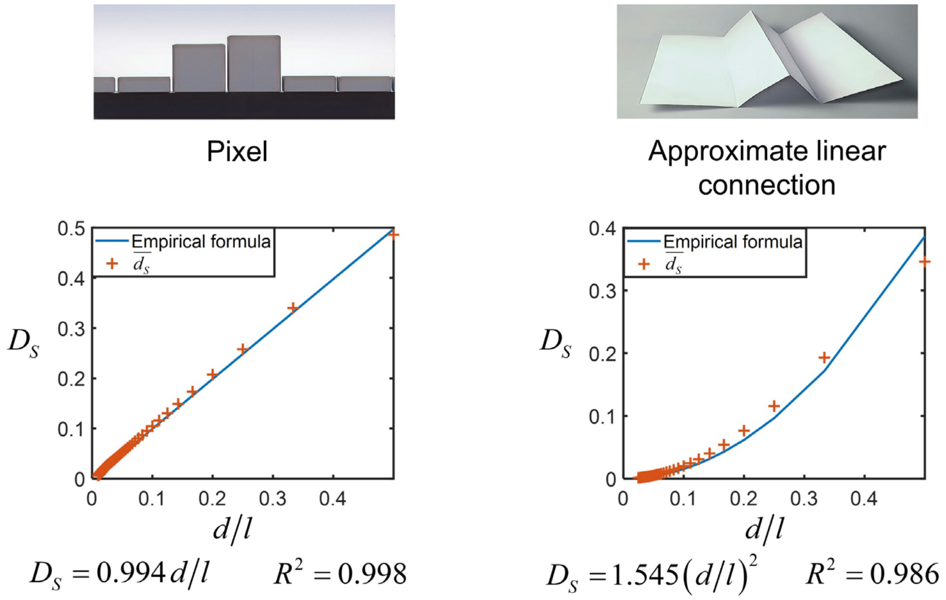

Supplementary Fig. 3 Fitting curve for the shape distortion of different types of 1D haptic devices.

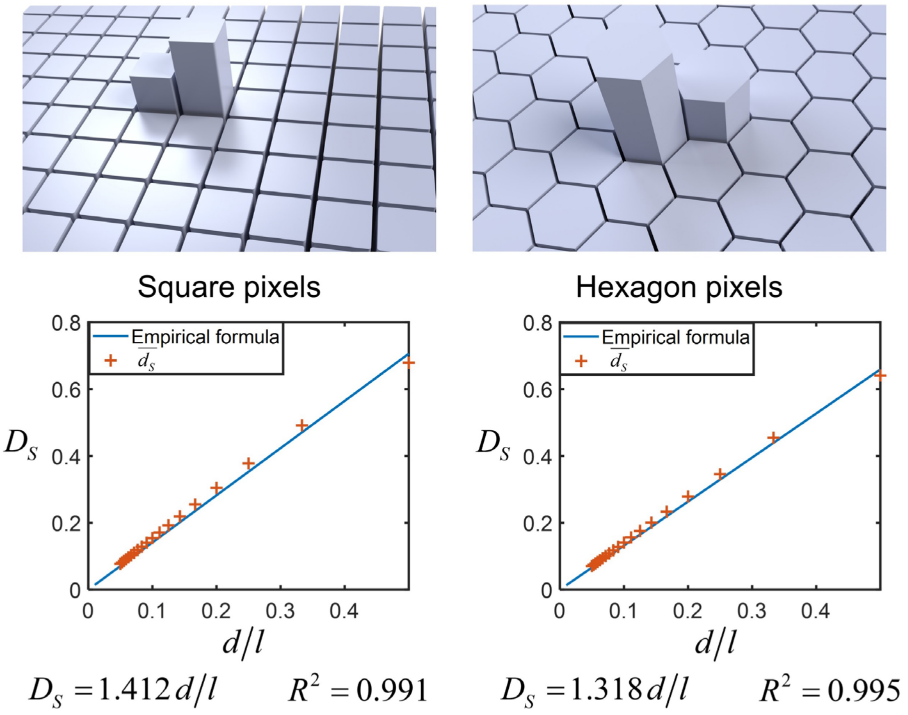

Supplementary Fig. 4 Fitting curve for the shape distortion of different pixel shape.

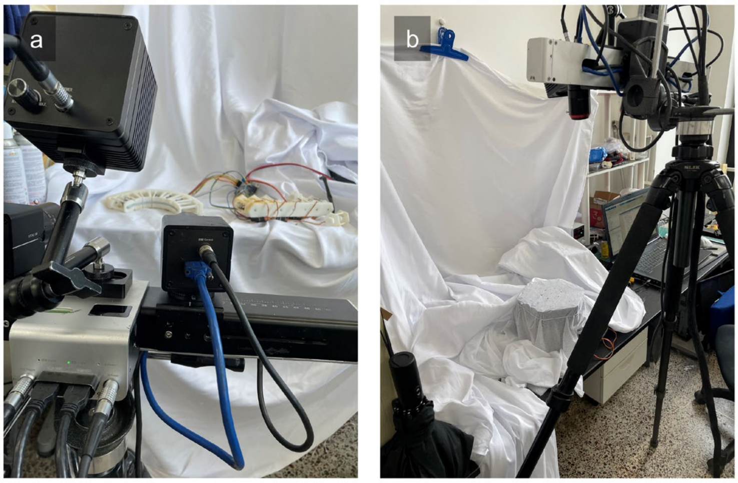

Supplementary Fig. 5 Digital Image Correlation (DIC) Method for measuring surface displacement. a Measurement of 1D curved Continuity Reinforcement Skeleton (CRS). **b** Measurement of a 2D CRS device.

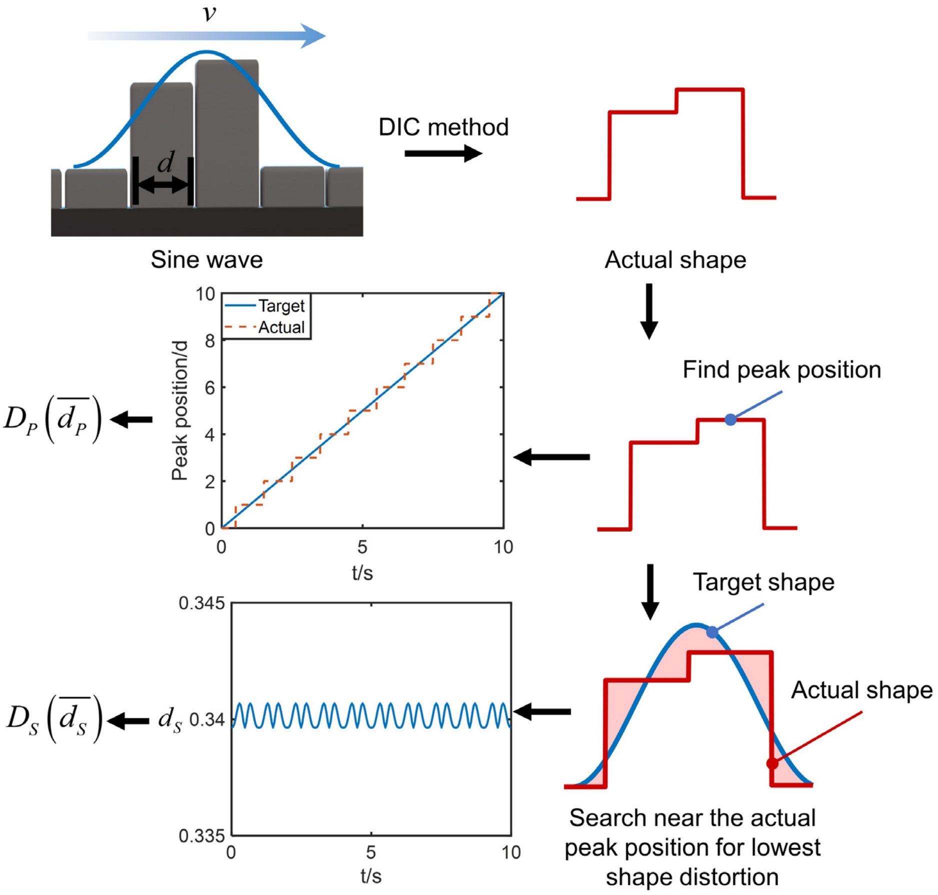

Supplementary Fig. 6 Experimental measurement procedure of D_P and D_S.

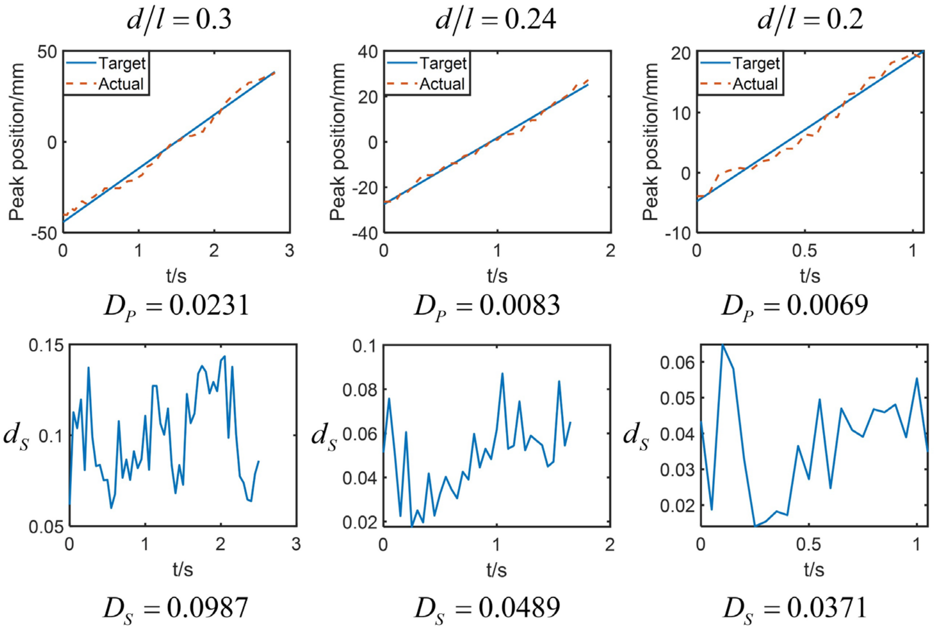

Supplementary Fig. 7 Experimentally measured D_P and D_S in 1D Continuity Reinforcement Skeleton (CRS) device under different d/l.

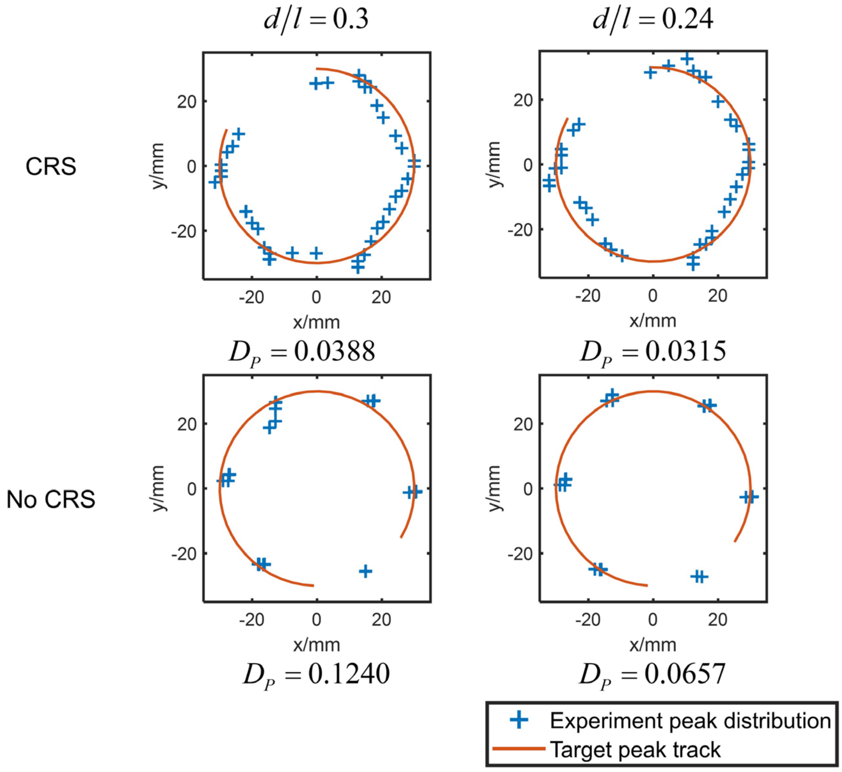

Supplementary Fig. 8 Peak distribution and target track of haptic device with 2D Continuity Reinforcement Skeleton (CRS) device or without CRS.

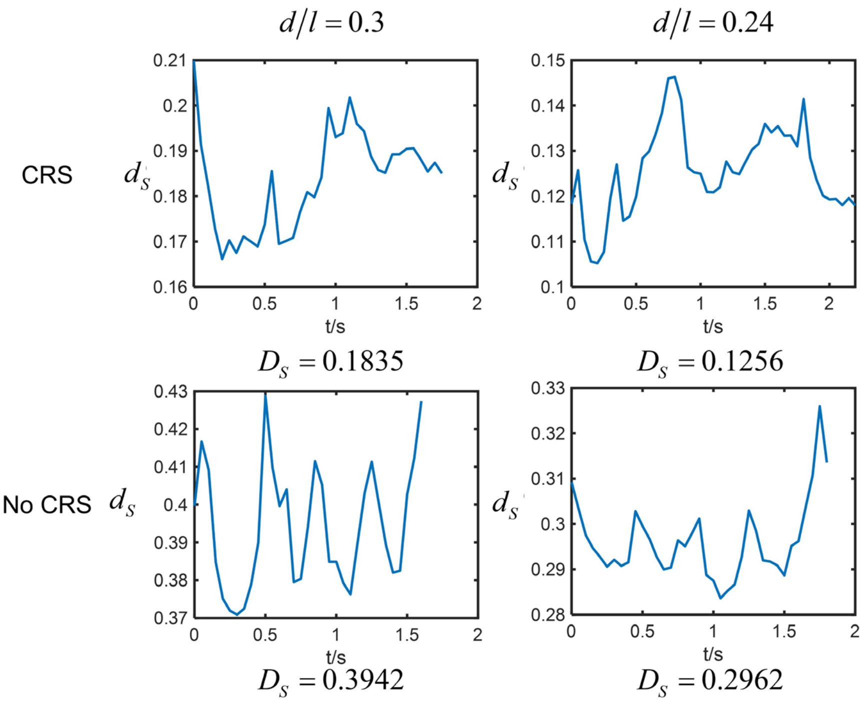

Supplementary Fig. 9 Instantaneous shape distortion of 2D haptic device with or without Continuity Reinforcement Skeleton (CRS).

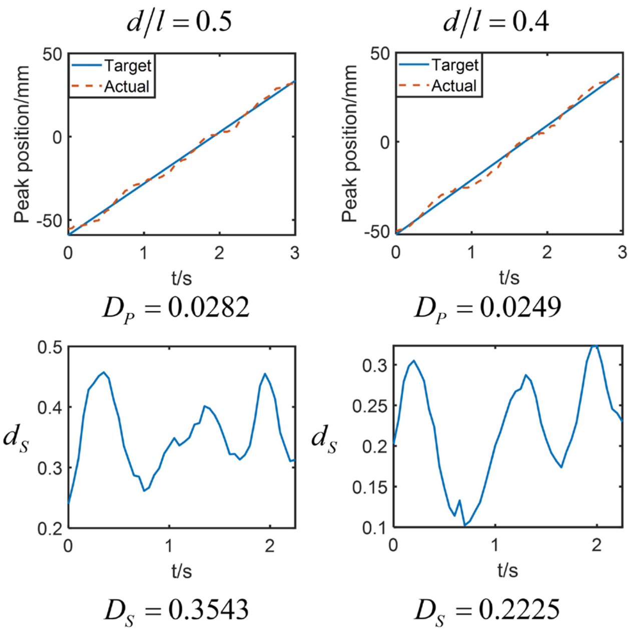

Supplementary Fig. 10 Distortion in the case of high d/l.

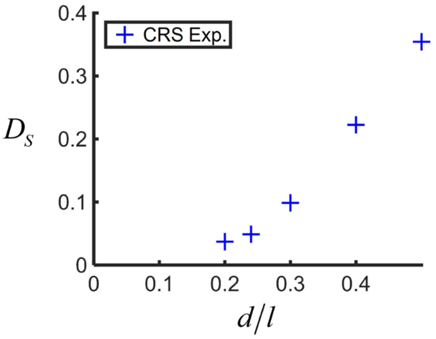

Supplementary Fig. 11 Experimental measurement of Continuity Reinforcement Skeleton (CRS) device's shape distortion as a function of d/l.

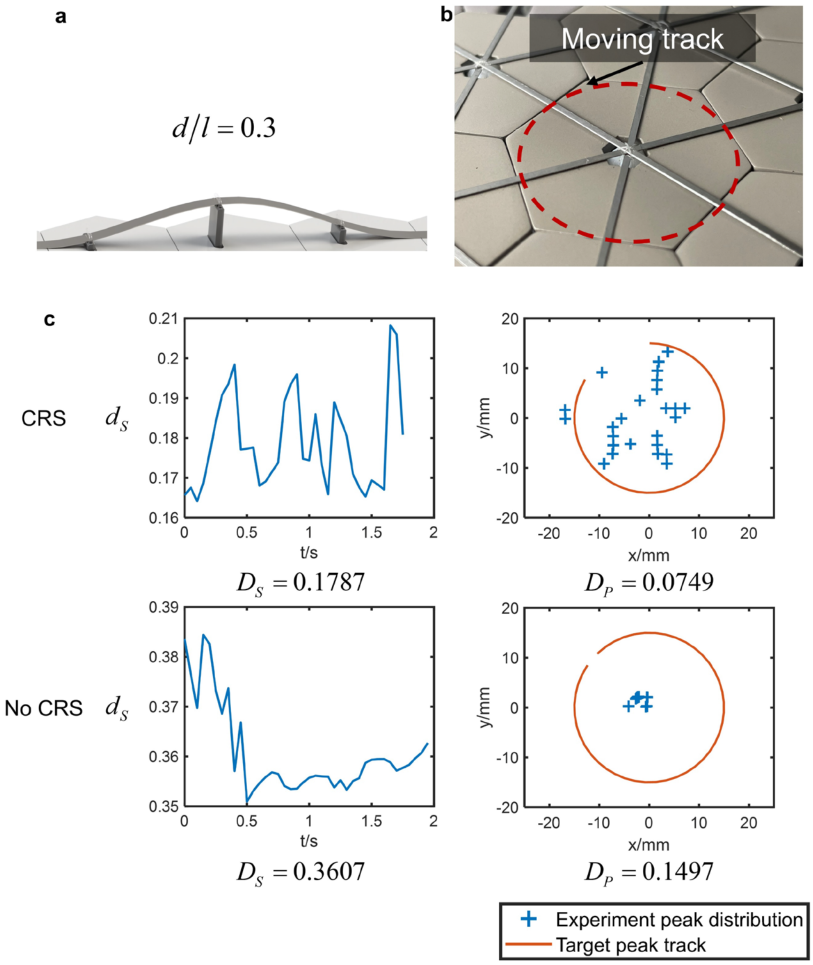

Supplementary Fig. 12 Testing of the the resolution limit of Continuity Reinforcement Skeleton (CRS). a Minimum wavelength for CRS. **b** Moving track to explore the resolution of CRS. **c** Distortion result of the CRS and no CRS at a radius of motion of 15 mm, $d/l = 0.3$.

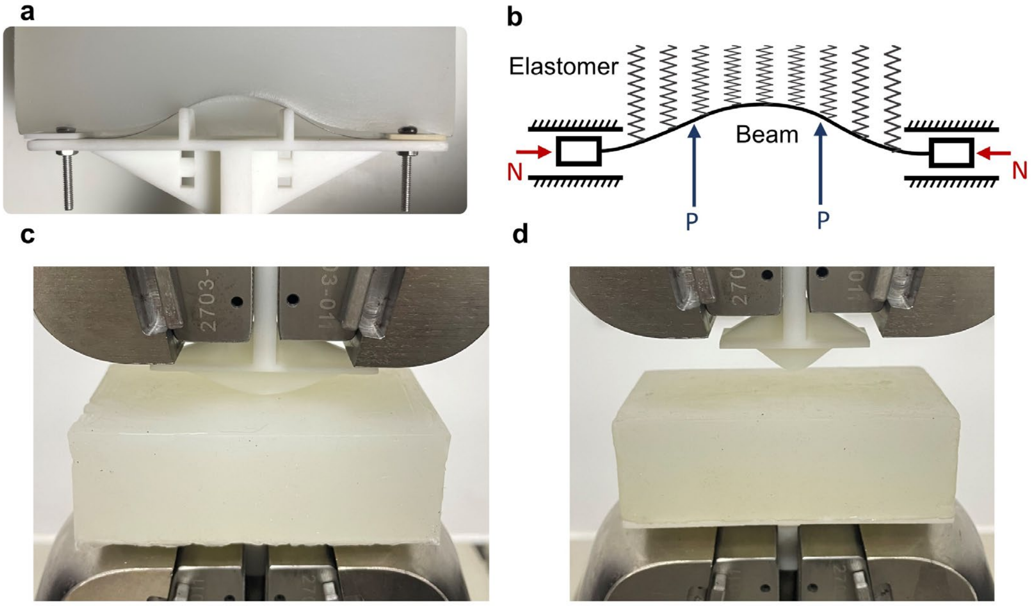

Supplementary Fig. 13 Mechanics of Continuity Reinforcement Skeleton (CRS). a CRS contact with elastomer. **b** Mechanics model for CRS. **c** Indentation test with an indenter of a wavelength 60mm in shape. **d** Indentation test with an indenter of a wavelength 30mm in shape.

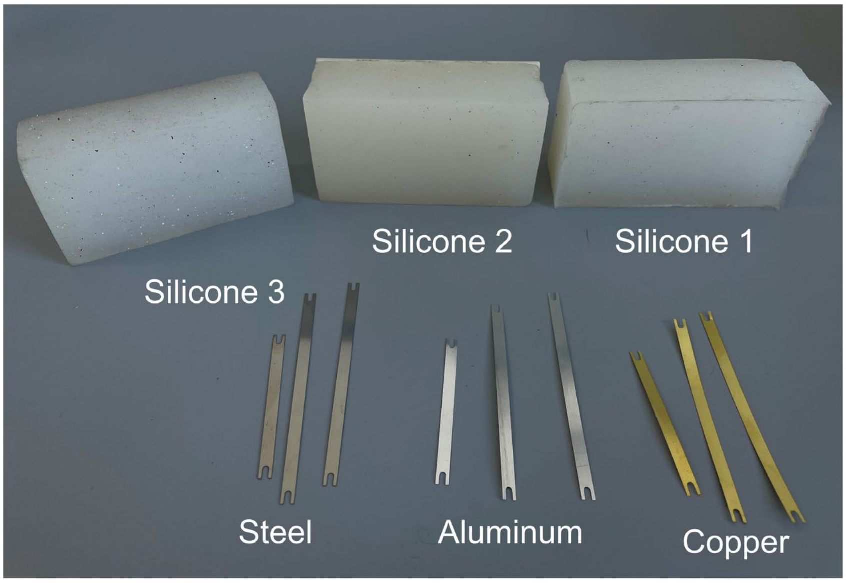

Supplementary Fig. 14 Materials and components used in collapse experiment.

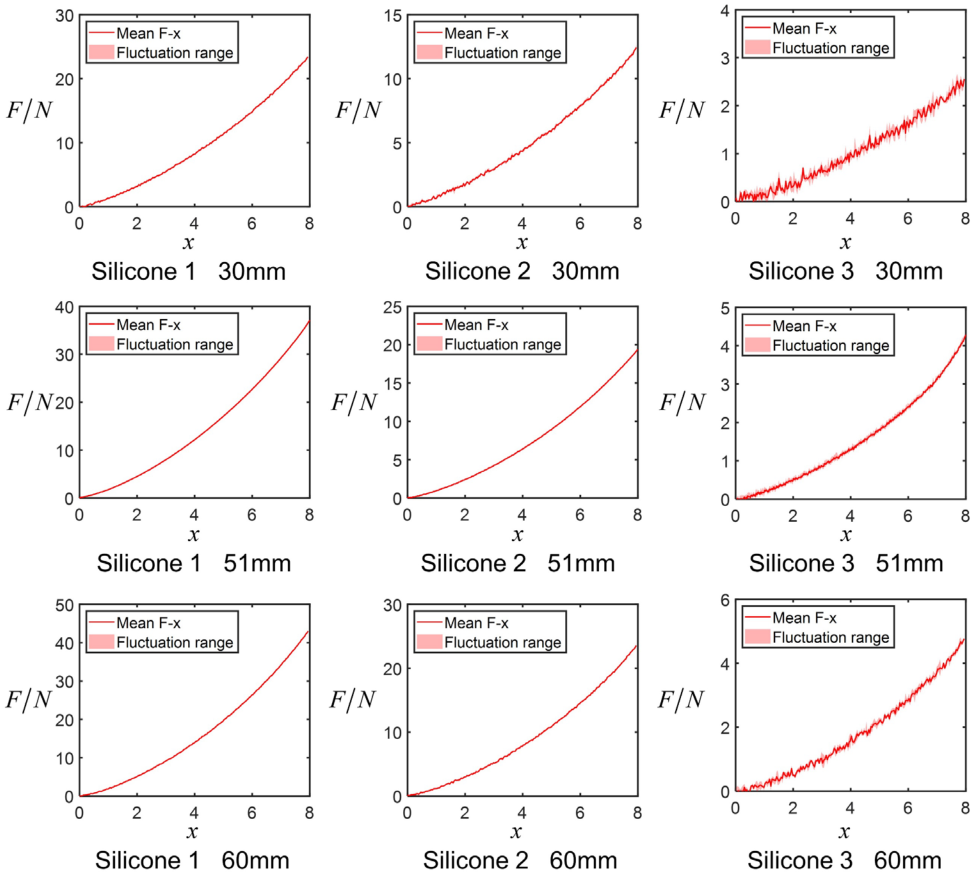

Supplementary Fig. 15 Force-displacement curves for different combinations of indenter wavelength and silicone.

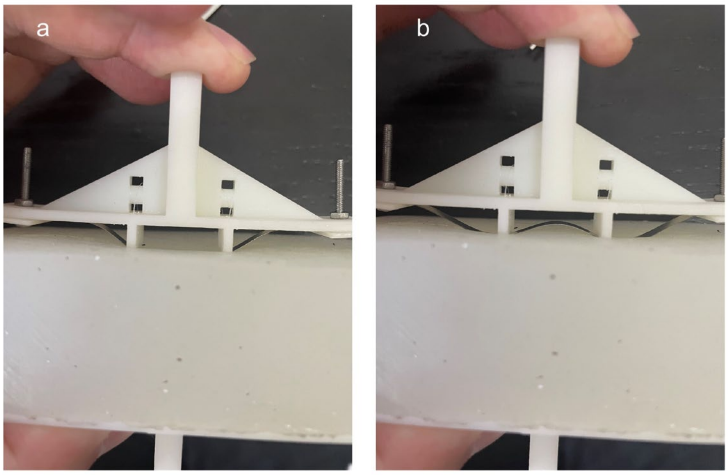

Supplementary Fig. 16 Collapse experiment at $\Delta \approx 1$. a Pressed in as a non-collapsed shape. **b** Pressed in as a collapsed shape.

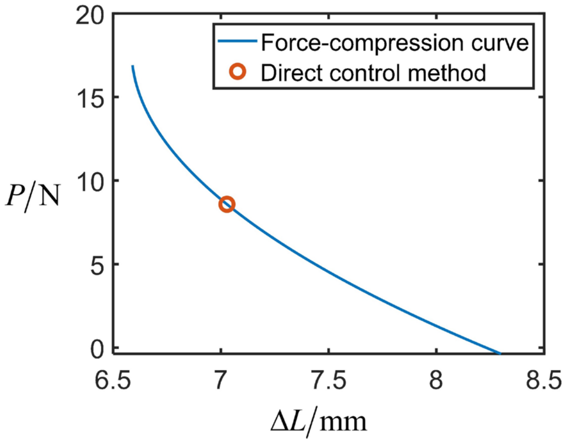

Supplementary Fig. 17 Relationship between pixel reaction force and boundary servo compression.

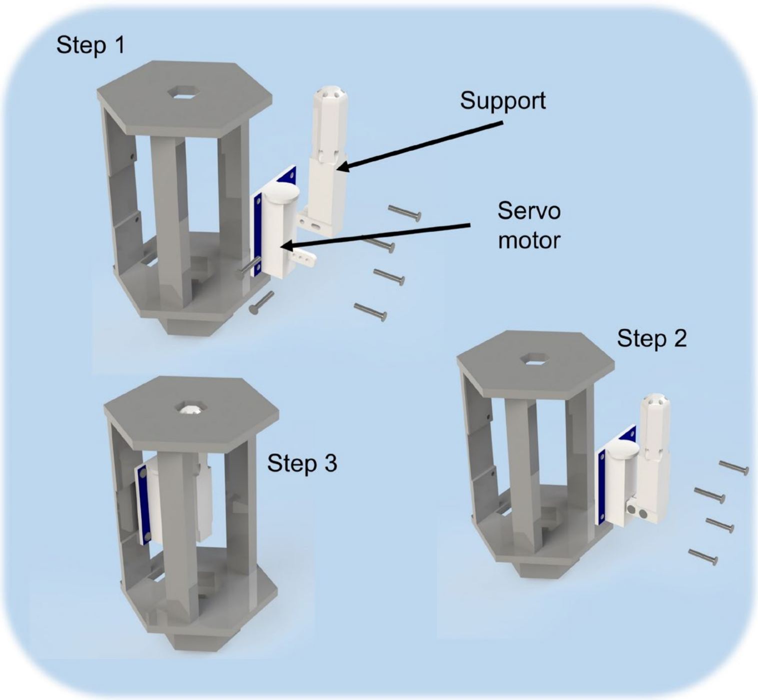

Supplementary Fig. 18 Assembling of inner unit.

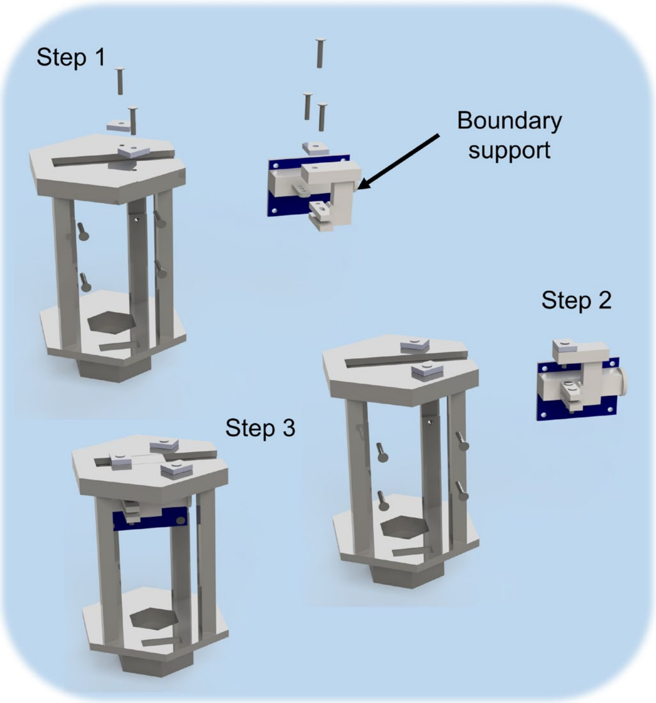

Supplementary Fig. 19 Assembling of boundary unit.

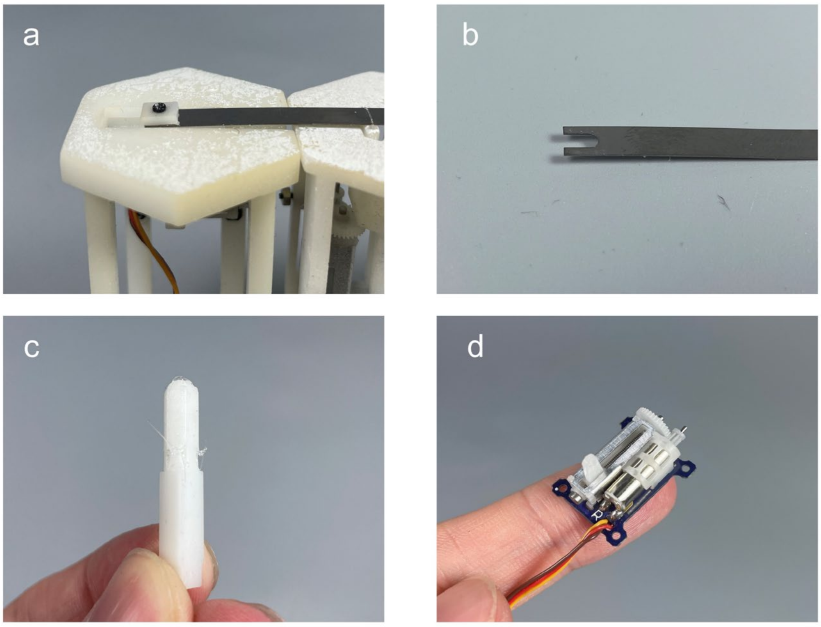

Supplementary Fig. 20 Details of Continuity Reinforcement Skeleton (CRS) components. **a** Connection of the boundary unit to the skeleton. **b** Design of the skeleton ends. **c** Attachment of fishing line to the supporter. **d** Linear servos used in the study.

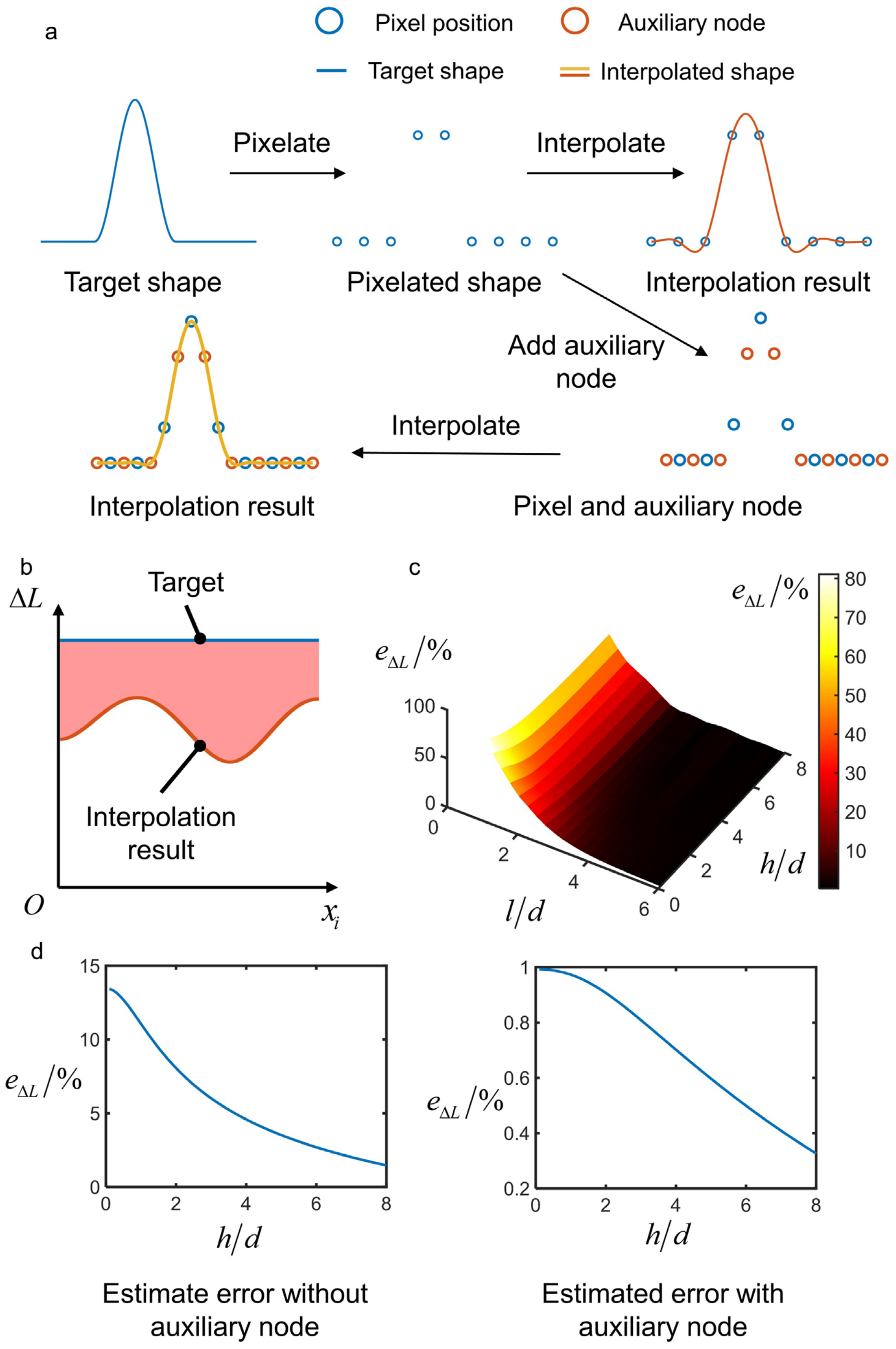

Supplementary Fig. 21 Interpolation control method of Continuity Reinforcement Skeleton (CRS). a Process to calculate the compression of CRS. **b** Relationship between the error of the estimation of ΔL and the peak position x_i. **c** Contour plot of $e_{\Delta L}$ in the l/d and h/d space. **d** Comparison of $e_{\Delta L} \sim h/d$ when $l/d = 3$ without and with an auxiliary node.

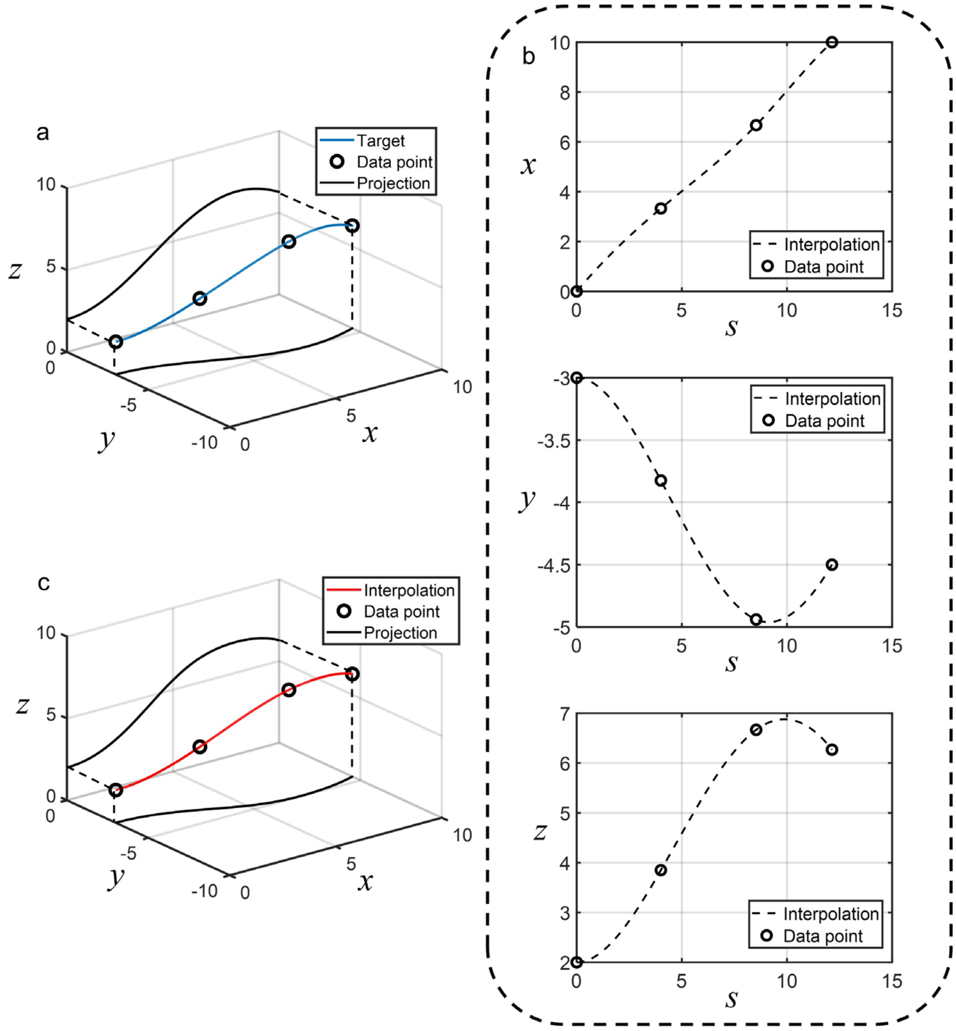

Supplementary Fig. 22 Interpolation in spatial data point. a Spatial curve and data point selection. **b** Seperate interpolation of x_i, y_i, and z_i. **c** Interpolation result for the spatial curve.

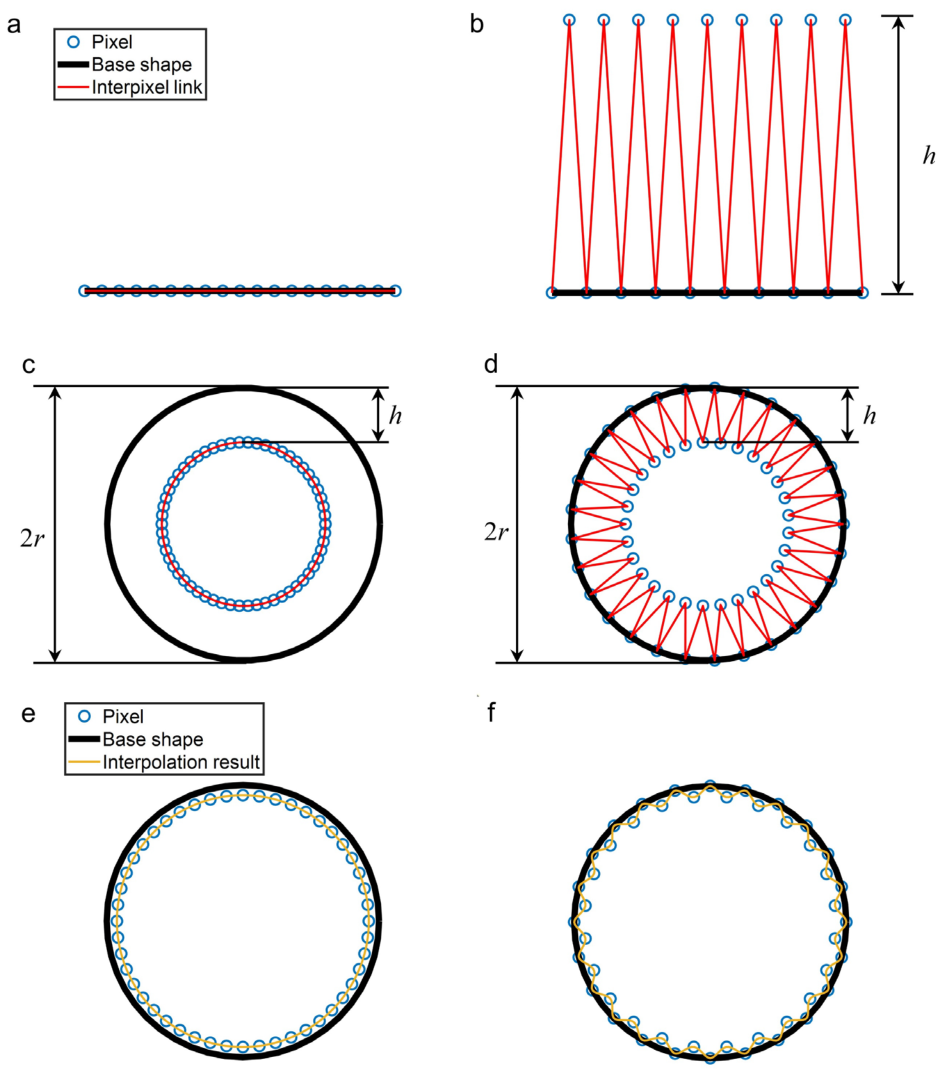

Supplementary Fig. 23 Travel Limit Schematic. **a** Shape of minimum length in straight base. **b** Shape of maximum length in straight base. **c** Shape of minimum length in circle base. **d** Shape of maximum length in circle base. **e** Minimum Continuity Reinforcement Skeleton (CRS) length estimation in wearable device. **f** Maximum CRS length estimation in wearable device.

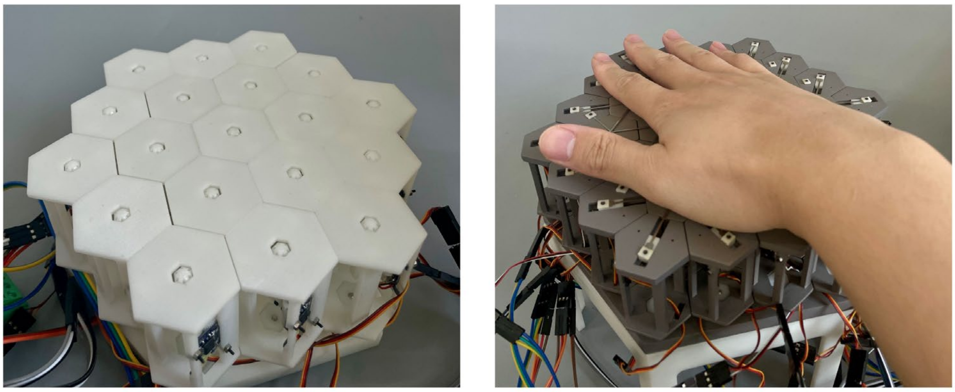

Supplementary Fig. 24 Pixel haptics and Continuity Reinforcement Skeleton haptic devices.

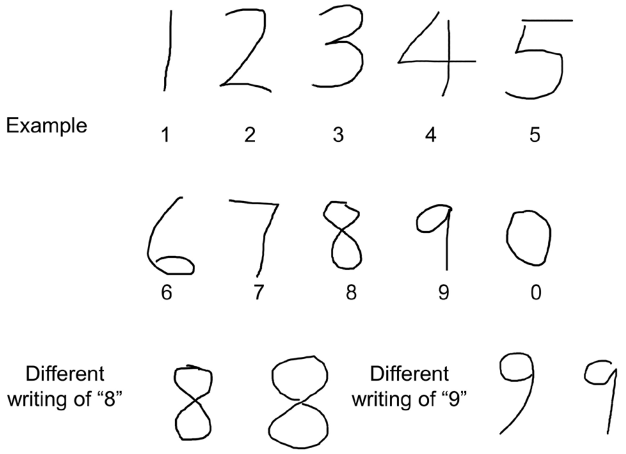

Supplementary Fig. 25 The trajectories of handwritten digits in the identification experiment.

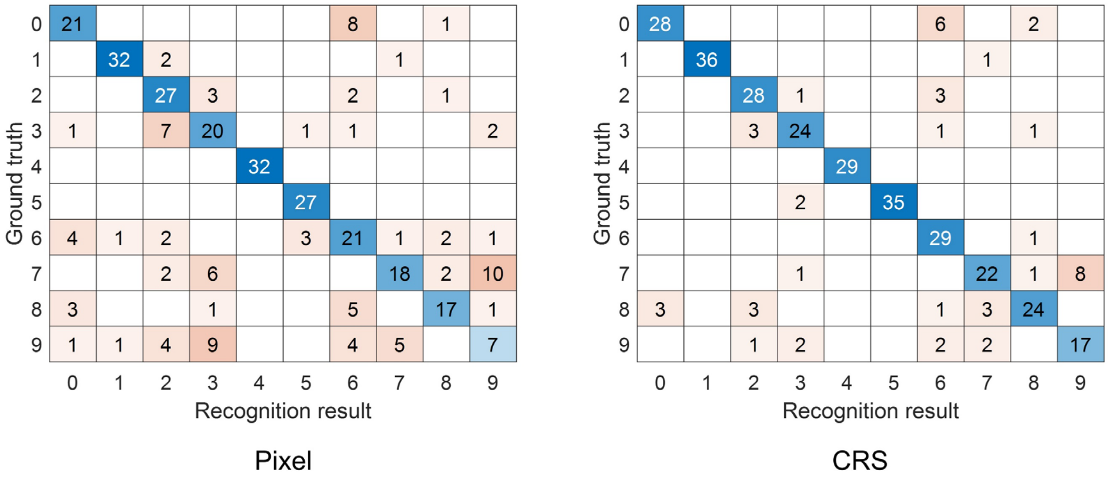

Supplementary Fig. 26 The confusion matrices of the pixel and Continuity Reinforcement Skeleton haptics in the digit identification.

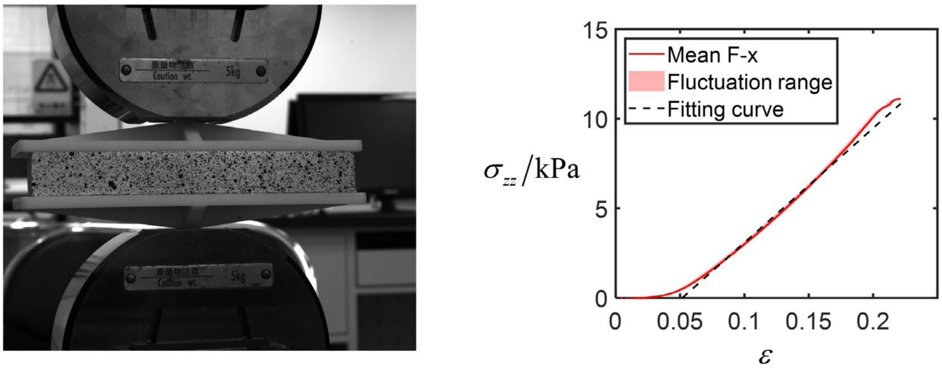

Supplementary Fig. 27 Uniaxial compression test of silicone.

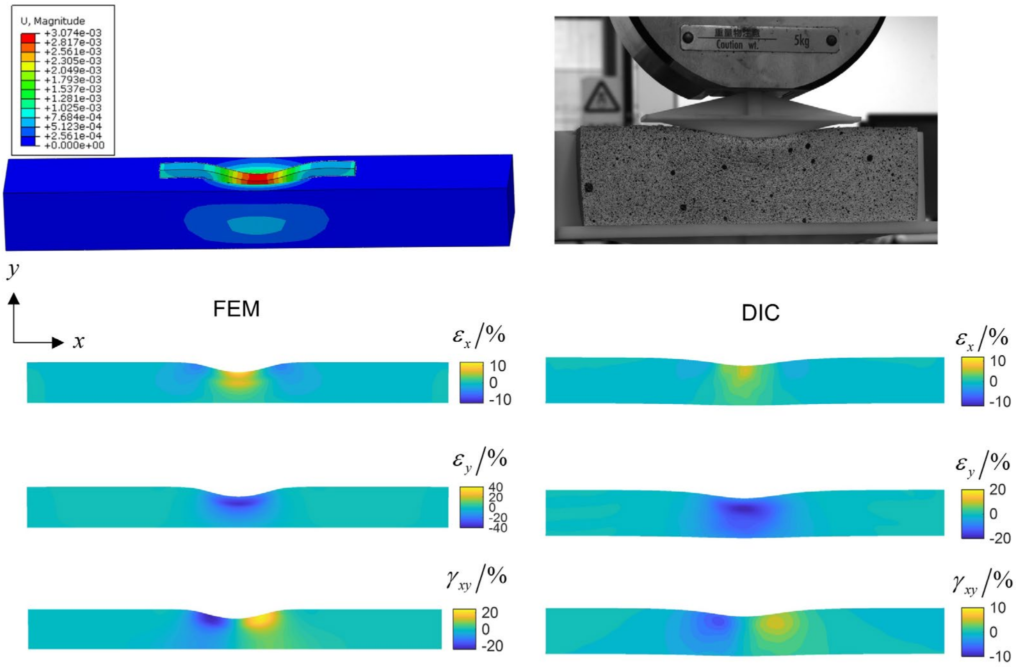

Supplementary Fig. 28 Numerically simulated and experimentally measured strain field under the skin. The simulations and measurements were obtained using the Finite Element Method and Digital Image Correlation methods, respectively.

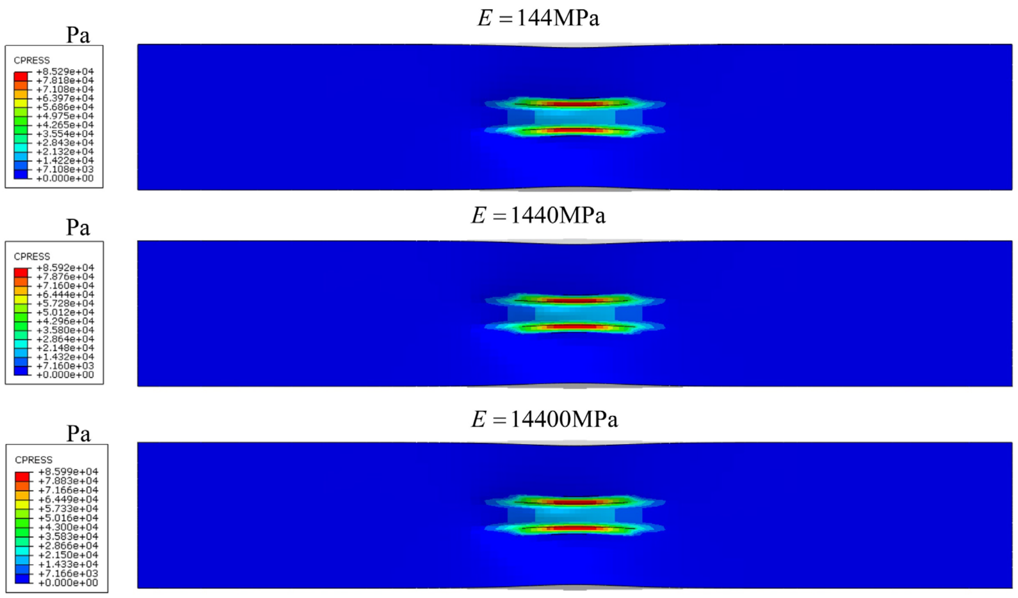

Supplementary Fig. 29 Pressure field on the skin generated by Continuity Reinforcement Skeleton.

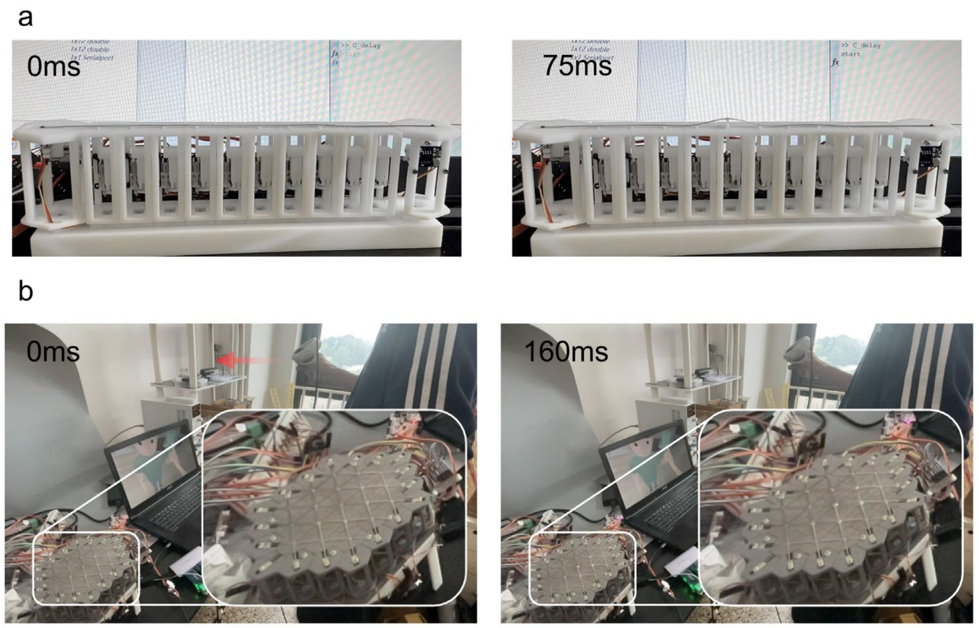

Supplementary Fig. 30 Latency measurement. a Latency measurement of the Continuity Reinforcement Skeleton device. **b** Latency measurement of the VR-haptic system.

Supplementary Table 1 Results of β obtained for different silicones at different wavelength indenters

Indenter wavelength\Silicone number	1	2	3
$l = 30\text{mm}$	1.95×10^{5}	1.05×10^{5}	2.18×10^{4}
$l = 51\text{mm}$	1.81×10^{5}	0.95×10^{5}	2.09×10^{4}
$l = 60\text{mm}$	1.80×10^{5}	0.99×10^{5}	2.00×10^{4}

Supplementary Table 2 Comparison of the three control methods

**Control method \ Item**	**Direct control method**	**Interpolation control method without auxiliary points**	**Interpolation control method with auxiliary points**
**Advantages**	Fast, accurate	Able to calculate compression if the heights of pixels are known	Accurate, able to calculate compression if the heights of pixels are known
**Disadvantages**	Require the equation of the CRS curve	Large error when displaying details of smaller wavelengths	Need additional height information for auxiliary nodes
