# Supplementary material for: Enhancing haptic continuity in virtual reality using a continuity reinforcement skeleton
Source: Nat Commun. 2025 Mar 27;16:2995. doi: 10.1038/s41467-025-58318-z (PMC11950344; doi:10.1038/s41467-025-58318-z)
Supplement: Supplementary file 2 — Description of Additional Supplementary Files [file 41467_2025_58318_MOESM2_ESM.docx]

**Inventory of Supplementary File**

**Supplementary Movie 1.**

**Continuous movement display of CRS.** We utilize a double sine wave generated by the 1D CRS to continuously control a ball with a size much smaller than the pixel pitch to stay at an arbitrary position between pixels.

**Supplementary Movie 2.**

**1D CRS device.** The 1D CRS produces continuous motion with low distortion (measured by the DIC method). Single-wave, two-wave isotropic motion, and two-wave anisotropic motion are demonstrated.

**Supplementary Movie 3.**

**CRS display effect.** The CRS interacting with the elastomer was established, and the different tactile sensations produced with and without the CRS were visualized by the displacement of the elastomer surface in the Y direction.

**Supplementary Movie 4.**

**2D CRS device.** The 2D CRS device can continuously demonstrate the movement of points, the spreading of ripples, and the gradual contact with a sphere. With the DIC method, the effect of CRS on haptic can be visualized.

**Supplementary Movie 5.**

**Visual-haptic integration virtual reality.** We built a VR scenario where the user can press the NPC’s hand. 2D CRS displays the corresponding tactile sensation in real time based on the depth and position of the press.

**Supplementary Movie 6.**

**CRS on curved surface.** CRS on curved surfaces was built to validate their wearability because most human body areas are curved.
